# Management Effectiveness of a Secondary Coniferous Forest for Landscape Appreciation and Psychological Restoration

**DOI:** 10.3390/ijerph14070800

**Published:** 2017-07-18

**Authors:** Norimasa Takayama, Akio Fujiwara, Haruo Saito, Masahiro Horiuchi

**Affiliations:** 1 Division of Forest Management, Forestry and Forest Products Research Institute in JAPAN, 1 Matsuno-sato, Tsukuba, Ibaraki 305-8687, Japan; 2 Fuji Iyashinomoroi Woodland Study Center, the University of Tokyo, 341-2 Yamanaka, Yamanakako Village, Minami-tsuru, Yamanashi 401-0501, Japan; akio@uf.a.u-tokyo.ac.jp (A.F.); haruo_s@uf.a.u-tokyo.ac.jp (H.S.); 3 Division of Human Environmental Science, Mt. Fuji Research Institute, 5597-1 Kami-yoshida, Fuji-yoshida, Yamanashi 403-0005, Japan; mhoriuchi@mfri.pref.yamanashi.jp

**Keywords:** Shinrin-yoku, forest management, profile of mood states, restorative outcome scale, positive and negative affect schedule, semantic differential method, perceived restorativeness scale

## Abstract

We investigated the influence of forest management on landscape appreciation and psychological restoration in on-site settings by exposing respondents to an unmanaged, dense coniferous (crowding) forest and a managed (thinned) coniferous forest; we set the two experimental settings in the forests of the Fuji Iyashinomoroi Woodland Study Center. The respondents were individually exposed to both settings while sitting for 15 min and were required to answer three questionnaires to analyze the psychological restorative effects before and after the experiment (feeling (the Profile of Mood States), affect (the Positive and Negative Affect Schedule), and subjective restorativeness (the Restorative Outcome Scale). To compare landscape appreciation, they were required to answer another two questionnaires only after the experiment, for scene appreciation (the semantic differential scale) and for the restorative properties of each environment (the Perceived Restorativeness Scale). Finally, we obtained these findings: (1) the respondents evaluated each forest environment highly differently and evaluated the thinned forest setting more positively; (2) the respondents’ impressions of the two physical environments did not appear to be accurately reflected in their evaluations; (3) forest environments have potential restorative effects whether or not they are managed, but these effects can be partially enhanced by managing the forests.

## 1. Introduction

Nearly 33 years have elapsed since the restorative effects of nature proposed by Ulrich [1] became a subject of scientific analysis, and since then, the issue has been studied from psychological perspectives compared with urban settings [2,3,4,5,6]. According to Haluza et al. [7], since the beginning of this century, the physiological and psychological restorative effects of forests have been investigated, primarily in developed countries where aging populations and declining birth rates have become serious issues. In Japan, since the concept of Shinrin-yoku (taking in a forest atmosphere or forest bathing) was proposed in 1982 [5], primarily in the second half of the 1990s, studies on the physiological and psychological restorative effects of forests progressed rapidly. Findings suggest that if citizens’ physical and mental health can be maintained by utilizing nature, including forests, medical expenses, and other costs could decrease, which would greatly benefit societies. For example, in the areas of medicine and physiology, Ohira et al. [8] clarified that Shinrin-yoku was effective in immune restoration, and Li et al. [9,10] reported that immune cells became more active after a three-day stay in the forest. Li et al. [11] also reported that the benefits of Shinrin-yoku were attributable not only to the differences in air quality but also to the overall environment compared with living in urban areas for the same duration. Ochiai et al. [12,13] reported that two hours of Shinrin-yoku reduced physiological parameters such as stress hormones, adrenaline, and blood pressure in middle-aged and elderly people. The Profile of Mood States (POMS; McNair et al. [14]) is generally applied to investigate improvements in feelings [15,16,17,18], and the State-Trait Anxiety Inventory (Spielberger et al. [19,20]) is used to study feelings of insecurity [21]. Furthermore, using other developed indices such as the Subjective Vitality Scale [22], the Restorative Outcome Scale (ROS; Korpela et al. [23,24]), and the Perceived Restorativeness Scale (PRS; Korpela and Hartig [25]; Hartig et al. [26,27]), it was clarified that Shinrin-yoku improved vigor [28,29,30] and was provided psychological restorativeness. Kobayashi et al. [31] conducted a large-scale field experiment known as forest therapy in 62 areas maintained for Shinrin-yoku. Moreover, a few researchers have examined the restorative effects of not only remote and suburban forests but also urban forests and confirmed considerable physiological and psychological effects [30,32]. In brief, researchers have confirmed the physiological and mental restorative effects of forest environments; previous research on Shinrin-yoku has made great contributions to the public health field by scientifically clarifying the restorative effects of nature.

However, in forest management, planning is necessary to enhance the health and recreational functions of forests; to use them continuously and comfortably, more information on how to manage the physical environments of forests. Regarding the relationship between the physical forest environment and Shinrin-yoku, Horiuchi et al. [33] observed that when individuals viewed real forest scenes, the cerebral oxygenated hemoglobin in the prefrontal area declined and their feelings improved more than they did when the participants were cut off from forest environments. Park et al. [34] reported a relationship between impressions and mood-restoring effects and physical features such as temperature or illumination. Takayama et al. [35] and Fujisawa and Takayama [36] investigated the effects of light in a forest and demonstrated that in the bright environment with sunlight passing through the trees, the blood volume in the participants’ brains decreased and their moods improved more than they did in the dark area in the same forest. Summarizing the results of the previous studies, an appropriate forecast of the forest environment is better for Shinrin-yoku. However, for effective forest management, it is important to understand each forest setting based on its intended use [37,38,39]. For instance, compared with remote artificial forests, suburban forests or forests near tourist areas require management to maintain biological diversity and at the same time satisfy users’ needs [37,38,39]. Accordingly, adequately managing urban forests with highly diverse uses such as Shinrin-yoku and other recreation, managers must be well informed about what aspects of management are required based on how the forests will be used.

The following studies have assessed the relationship between people’s evaluations of forests and forest management methods could be helpful. Buhyoff and Leuschner [40] showed that forest favorability ratings decreased suddenly and sharply when damage from pinewood nematodes exceeded 10%; Takahashi et al. [41] suggested that people’s evaluations of forests vary depending on the mixes of hardwood and softwood; and Oishi et al. [42], Takayama et al. [43], and Takayama et al. [44] reported on the relationship between people’s assessments of the comfort of forest environments and tree density. Other researchers have also investigated the effects of types and numbers of trees, tree density, planting patterns, presence or absence of fallen trees, and other factors on people’s evaluations of landscape beauty.

Meanwhile, one method of controlling forest density is known as thinning. Thinning improves light environment and soil, which encourages the growth of the remaining trees even when growth is still dense. In addition, thinning is not only useful for trees but also highly related to users’ forest experiences. However, few studies have investigated the relationship between thinning and perceptions of forest beauty [45,46,47]. Daniel [48] reported that forest visitors in general prefer managed forests in which trees grow well to entirely unmanaged natural forests, and Edwards et al. [49] highlighted that thinning not only maintains forests in good condition, but also improves people’s evaluations of forests’ scenic beauty. However, it is necessary to further organize the knowledge about the impact on users’ evaluations of forests’ esthetic value of controlling stand age, forest type, thinning, and other management factors. Studies have been performed that considered the connections between forest management and recreation. Brunson and Shelby [50] reported that the landscape quality of forests and recreational activities are closely linked. Kunisaki and Imada [51] suggested that thinning is essential for effectively managing forest density along roads and sidewalks to increase user satisfaction; researchers at the Gifu Prefectural Research Institute for Forests [52] found that visitor ratings increase when trees are maintained approximately 20 m from either side of forest roads; and Oku [53] studied the ease of performing forest activities including analyzing the management of the physical environment based on intended use.

These studies lead to a number of questions. How does our evaluation of forests change depending on the degree of thinning in the forest, and how do these factors relate to psychological and physiological restoration? Unfortunately, there are only a few previous studies such as the one by Oishi et al. [15], and the data on the subject are still insufficient.

Meanwhile, in urban forests or those in resort areas that are already in use for forest recreation, some facilities are reasonably managed, unlike in remote forests where care has been abandoned. Health consciousness has been recently promoted, and forest managers should aim to sustainably provide Shinrin-yoku experiences that increase the restorative effects of forests on visitors, which should improve users’ general evaluations of forests. Thus, managers need to be aware of the best criteria for frequency of forest thinning, weeding, and pruning based on their intended goals.

Currently, as we mentioned above, the related research is insufficient, and hence forest administrators must make arbitrary choices about thinning based on their own judgment and experience without scientific evidence. In this context, if users cannot efficiently realize the benefits of thinning because of the lack of information on forest management and on the psychological and physiological restorative effects of forest environments, forest managers and users both miss excellent opportunities to take advantage of forests.

**Figure 1:**
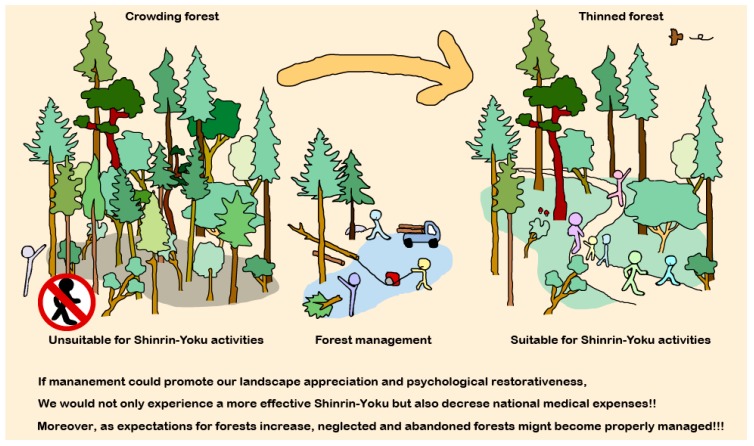
Forest management and our health.

However, if there is scientific evidence on how to improve and maintain the physical and psychological restorative features of forests, managers will gain objective guidelines for managing forest environments and for increasing the effects of Shinrin-yoku. In turn, users will enjoy their Shinrin-yoku experiences in more comfortable forest environments, and as noted earlier, if the mind and body can be restored by Shinrin-yoku, the increasing medical expenses that accompany aging populations could begin to decrease, which will significantly contribute to public health (Figure 1). Therefore, we believed that it would be most effective to understand whether forest maintenance affects the restorative features of individual forests. Beginning with such an investigation would give us fundamental information on the relationships between forest improvement, their restorative effects, and users’ appraisals of similar forest environments.

For this research, we planned an experiment in an urban forest located in a suburban summer resort. The goal of the research was to determine how thinning influenced users’ impressions and evaluations of the forest and the restorative effects of the forest environment.

## 2. Materials and Methods

### 2.1. Research Design

We conducted our experiment from 26 to 29 July 2014, when the average temperature and relative humidity were 21.7 °C and 83.7%, respectively (Table 1). 

**Figure 2:**
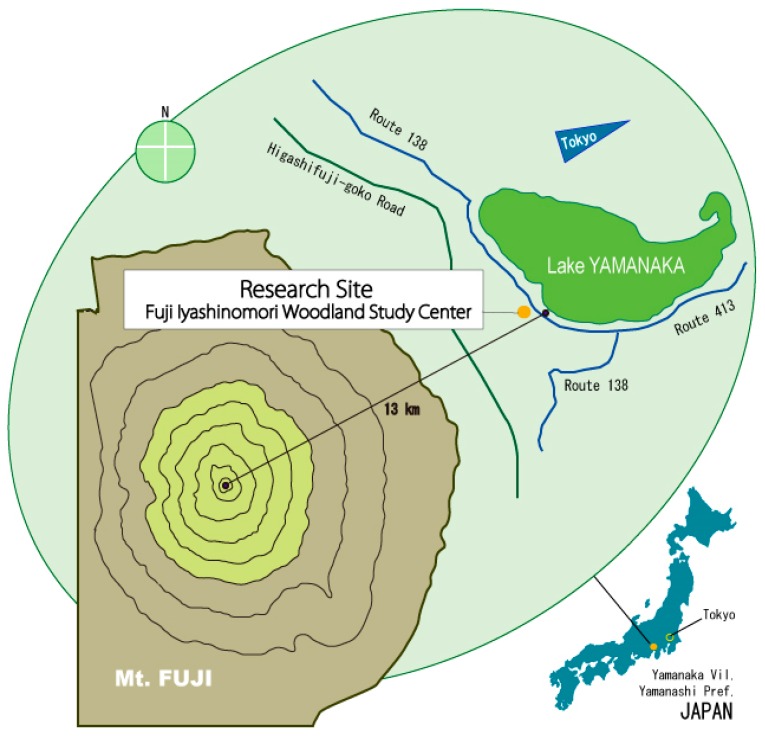
Locations of the research sites.

**Figure 3:**
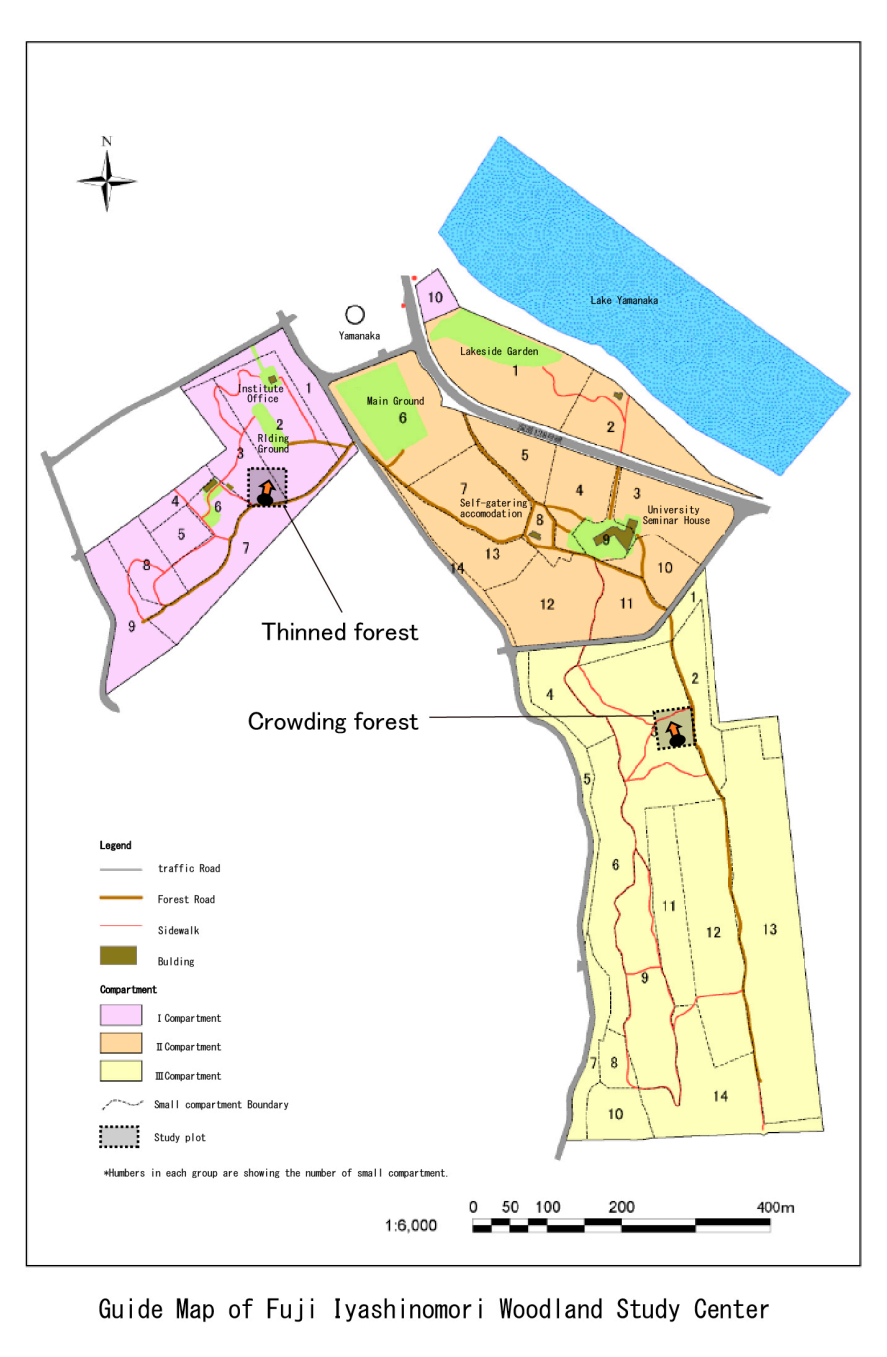
The experimental plots. The authors modified this figure from the figure on the Fuji Iyashinomori Woodland Study Center’s website (http://www.uf.a.u-tokyo.ac.jp/fuji).

We chose the Fuji Iyashinomori Woodland Study Center as the study area, an experimental forest associated with the University of Tokyo located in the Yamanakako village around Mt. Fuji. Figure 2 shows the two experimental plots, a crowded, unmanaged, dense forest (crowding forest) and a thinned, well-managed forest (thinned forest); Figure 3 and Table 1 show that each plot measured 2500 m^2^ (50 × 50 m. The experimental forests were manmade and primarily consisted of approximately 80-year-old larches *(Larix kaempferi*); however, the amounts of hardwood were increasing, and both plots were becoming mixed-type forests. In 2013, one year before the experiment, the shrubs and young trees were cut and removed from one of the two plots. As a result, the tree crown was considerably thinned, and the forest was in good condition (Table 2, right side); in contrast, in the unthinned forest, the crown was crowded (Table 2, left side). 

Table 2 also shows the distribution of vegetation used for the two experimental stimuli; the first percentage is for the crowding forest, and the percentages in parentheses are for the thinned forest: 66.5% (66.3%) Japanese larch (*Larix kaempferi*), 7.0% (10.1%) giant dogwood (*Cornus controversa*), 7.3% (7.3%) Japanese red pine (*Pinus densiflora*), 0.0% (6.4%) fir trees (*Abies*), and 19.2% (9.7%) other vegetation depending on the breast height of the basal area in the crowding forest.

### 2.2. Respondents

Seventeen male participants participated in this study (Table 3), and for the study, we measured physiological outcomes for different study designs. We conducted the experiment twice with one of the participants, because we failed to obtain physiological outcomes for him. We acknowledge that this might have been an exceptional design; however, we added the data for further analysis because even the same participant’s psychological responses can differ depending on their feelings. Thus, the numbers of total data set were 18 (=18 respondents). We chose to investigate working males because they appear to experience the most social stress but are rarely participants in this type of research. All the respondents lived in Yamanakako village and its neighboring area, where the research sites were located. Because the forest area of the Fuji Iyashinomori Woodland Study Center is not usually open to the public, all respondents except for four who were center staff were entering the forest for the first time. We could not assess the respondents’ forest preferences or expertise, but by profession, none worked in forest management except for the four staff members. All respondents had no history of cardiovascular diseases or mental illnesses, and none were taking any medications that could affect psychological responses. Following a detailed description and explanation of the study procedures and the possible risks and benefits of participation, each respondent signed an informed consent form. We randomly assigned the respondents to one of two groups, Group A or Group B, of nine and asked them to abstain from consuming caffeinated beverages for 12 h and to abstain from strenuous exercise or consuming alcohol for a minimum of 24 h before the experiment. All procedures applied in the present research were approved by the ethics committee of the Mt. Fuji Research Institute (ECHE-032012) and were performed in accordance with the guidelines of the Declaration of Helsinki.

### 2.3. Environmental Measurements

We measured the physical environment in order to grasp the physical conditions of each setting. We measured temperature and relative humidity, wind velocity, and radiant heat using a portable amenity meter (AM-101; Kyoto Electronic Manufacturing, Co., Ltd., Kyoto, Japan) and measured illumination intensity using an illuminometer (T-10; Konica-Minolta, Tokyo, Japan). We also measured sound pressure using a sound level meter (Center322; Center Technology Corp., New Taipei, Taiwan). We measured these parameters throughout the 15-min experiments every 5 min for each respondent.

### 2.4. Questionnaires

**Figure 4:**
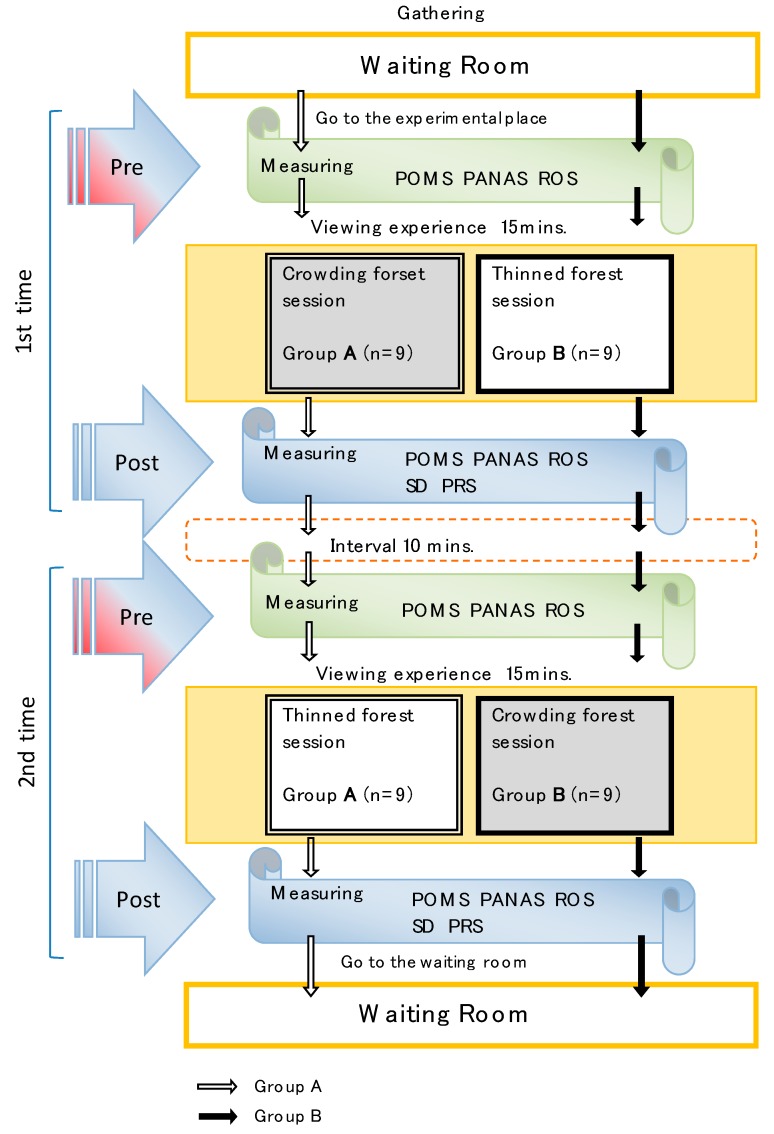
Experimental procedure. The same procedure was used in the both thinned and crowding forest sessions.

We used the following five psychological questionnaires. Figure 4 shows how we administered the questionnaires.

#### 2.4.1. POMS

The POMS [14] is a well-established, factor-based, and analytically derived measure of feelings, and its reliability and validity have been well documented. It measures the following six mood states: tension–anxiety (T–A), depression–dejection (D), anger–hostility (A–H), vigor (V), fatigue (F), and confusion (C). We chose the Brief Form Japanese Version of the scale [54] for our experiment. Each subscale has five items, and thus, there is a total of 30 items; we used the raw scores for the statistical analyses.

#### 2.4.2. Positive and Negative Affect Schedule

The PANAS [55,56] measures positive (PA) and negative (NA) affect with 10 items for each. We used the Japanese version of the scale, which has 16 items, eight each for PA and NA, developed by Sato and Yasuda [57]. The items are measured on a seven-point Likert scale, and we used the respondents’ total scores for the statistical analyses.

#### 2.4.3. ROS

The ROS [23] was based on previous measures and findings regarding restorative outcomes [58,59,60], and its reliability was confirmed in previous studies [23,24]. It uses six items to investigate restorative, emotional, and cognitive outcomes in a given environment; each item is rated on a seven-point Likert scale (ranging from 1: not at all to 7: completely). For this research, we used the ROS-J translated into Japanese and checked for reliability and validity by Fujisawa and Takayama [61] and used the total score of the six items for the statistical analyses.

#### 2.4.4. Semantic Differential Scale

We used the standard deviation (SD) scale to clarify the influence of the different environments. The scale was initially developed by Osgood [62], and it permits qualitatively evaluating the influence of environments using adjectives and adjective–verb pairs with words that have opposite meanings; the items are rated on seven-point Likert scales. Park et al. [34] and Takayama et al. [44] confirmed the use of this scale in previous studies, and for this study, we used 25 adjective and adjective verb pairs for the respondents to use to evaluate the forest environments.

#### 2.4.5. PRS

We used the PRS to investigate the restorative properties of each environment, specifically, the scale developed by Hartig [63] and edited based on Kaplan and Kaplan’s Attention Restoration Theory [64]. Shibata et al. [65] translated the PRS into a Japanese version that comprises 26 items measured on 11-point Likert scales and can measure the extent to which a particular environment restores mental alertness, including “being away”, “fascination”, “coherence”, “scope”, and “compatibility”. In addition, this version measures “familiarity” and “preference”.

### 2.5. Procedure

Each respondent participated in two study sessions, one each in the crowding and thinned forest settings (Figure 4); we seated the men in comfortable chairs for the duration of each session. We hung cloth sheets on both sides of the respondent for the entire duration of the study and hung another sheet in front of each; we revealed each forest view by opening the cloth in front of the respondent.

We measured each respondent’s psychological responses to both the crowding and thinned forest settings. Specifically, we administered the POMS, PANAS, and ROS both before and after the 15-min experimental sessions and administered the SD and PRS after each session. Before each experimental session, we had the respondents sit upright in a comfortable chair to complete the first three questionnaires and then instructed them to view the scenery in either the crowding forest or the thinned one for 15 min. After each 15 min, we administered all five questionnaires. After both experimental sessions, we conducted additional experiments (all data are available as Supplementary Materials).

### 2.6. Data Analysis

First, we used unpaired *t*-tests to compare the respondents’ results for the crowding versus thinned forests and then aggregated the data for all study indices and calculated averages and SDs. We conducted parametric two-way repeated-measure ANOVAs to analyze the interactions and the (simple) main effects of the POMS, PANAS, and ROS scores as before-and-after indicators of the psychological restorative effects of exposure to the crowding versus thinned forests.

In contrast, for the SD scale and the PRS, we used paired *t*-tests to compare the results for the crowding and thinned forests to investigate the changes in the respondents’ evaluations of the different forest environments. In addition, every 15 min, we recorded each measurement indicator and compared the results, also using paired *t*-tests. We conducted all statistical analyses using Excel statistical software (Ekuseru-Toukei 2015; Social Survey Research Information Co., Ltd., Tokyo, Japan), and for each analysis, we calculated effect size “*r”*: small = 0.10; medium = 0.30; and large = 0.50, and “η^2^”: small = 0.01; medium = 0.06; and large = 0.14.

## 3. Results

### 3.1. Physical Environment

Table 4 shows the results of the physical environment measurements of the crowding and thinned study area forests; we found no statistically significant differences in temperature or radiant heat. However, for humidity and wind speed, we did observe statistically significant differences between the two settings; in the thinned forest, the humidity was higher (*p* < 0.01) and the wind was faster than in the crowding forest. We also compared the illuminance and the sound pressure, and the results showed that the illuminance in the thinned forest was nearly twice as great (*p* < 0.01) and the sound pressure was slightly higher (*p* < 0.01) than in the crowding forest.

### 3.2. Impression and Restorative Trait Evaluation

#### 3.2.1. SD (Appreciation for the Environment)

Table 5 shows the results of comparing the respondents’ impressions of the crowding and thinned forests. We confirmed statistically significant differences in the following nine scales: “bright–dark” (*p* < 0.01), “open–closed” (*p* < 0.05), “comfortable–uncomfortable” (*p* < 0.05), “ugly–beautiful” (*p* < 0.01), “dull–refreshing” (*p* < 0.05), “orderly–chaotic” (*p* < 0.01), “insecure–secure” (*p* < 0.05), “thin–thick” (*p* < 0.01), and “healthy–unhealthy” (*p* < 0.05).

In other words, the respondents appreciated that the thinned forest was bright and open, and they rated it as comfortable, beautiful, refreshing, orderly, deserted, and healthy, which could result in peace of mind. In addition, we found marginally significant differences (*p* < 0.1) for “friendly–unfriendly,” “non-enjoyable–enjoyable,” and “restless–calm.” The results show that the respondents found the thinned forest environment more friendly, favorable, and calm.

#### 3.2.2. PRS (Restorative Traits of Environments)

Table 6 shows the results of the respondents’ comparing the restorative traits of the crowding versus thinned forests. The comparisons showed that of the PRS’s restorative trait indices, the respondents rated “compatibility” statistically higher (*p* < 0.05) in the thinned forest than in the crowding forest and rated “coherence” and “preference” marginally significantly higher (*p* < 0.1). However, regarding the other indices (“being away”, “fascination”, “coherence”, “scope”, and “familiarity”), we observed no statistically significant differences between the crowding and thinned forest settings.

### 3.3. Psychological Restorative Effects

#### 3.3.1. POMS (Feeling)

We considered two types of psychological restorative effects, the effects of the different forest environments (crowding or thinned) and the effects of the exposure to the different environments (before versus after). Then, we set crowding versus thinned forest and before and after exposure to the forest environment as two factors and used a two-way repeated-measures ANOVA to compare the changes in the POMS scores and to analyze the interactions between factors and the (simple) main effects using the POMS data (Table 7 and Table 8).

For all six POMS indicators—tension–anxiety (T–A), depression–dejection (D), anger–hostility (A–H), vigor (V), fatigue (F), and confusion (C)—we found no interactions between thinning versus crowding or in before and after exposure to the forest environment. Furthermore, we analyzed the (simple) main effects of the condition and time differences. For condition differences, we found no effects for any of the indicators; on the contrary, for the time differences, we confirmed statistically significant effects for T–A and C (*p* < 0.01) and a marginally significant effect for V (*p* < 0.05; Table 7).

The results for the Bonferroni comparisons showed that despite the crowding forest setting, the scores for T–A and C decreased significantly (*p* < 0.05) and the score for V significantly increased (*p* < 0.01) after the men were exposed to the forests; that is, feelings improved after exposure to the crowding forest. However, for the thinned forest, the scores for T–A (*p* < 0.01) and C (*p* < 0.05) also decreased significantly and that for V also increased significantly (*p* < 0.01). The F score also declined marginally significantly (*p* < 0.1). Based on these results, we suggest that feelings also improved in the thinned forest setting.

In addition, before the experiment, no indicators showed statistically significant differences between conditions, whereas after the experiment, the D score for the thinned forest was significantly lower (*p* < 0.05) than that for the crowding forest.

#### 3.3.2. PANAS (Affect)

We conducted two-way factorial ANOVAs of the PANAS data, using condition differences and time differences as the two factors (Table 9 and Table 10).

The results showed no interactions between the condition and time differences between the two indicators NA and PA. On the contrary, for the (simple) main effects, although we could not find statistically significant differences in the main effects for the crowding versus thinned forest settings, in the comparisons of before and after the experiment, we found a significant effect for time differences for NA (*p* < 0.05; Table 9).

Based on the results of the multiple comparison tests, in the crowding forest, we observed a decrease in NA between before and after the experiment (*p* < 0.01; Table 10). There was also a marginally significant difference in the NA (*p* < 0.1) in the comparison between before and after exposure to the stimuli, and this tendency was more pronounced with exposure to the thinned forest.

#### 3.3.3. ROS (Subjective Restorativeness)

For the ROS, which we administered to investigate the subjective restorative aspects of the two forest settings, we conducted two-way factorial ANOVAs using the condition and time differences as the two factors, similar to what we did with the POMS and PANAS (Table 11 and Table 12).

We found no interactions between the condition and time differences, although regarding the (simple) main effects, we observed a marginally significant effect (*p* < 0.1) between before and after the experiment; however, regarding the differences in the environments, we found no significant effect of the crowding versus thinned forest settings (Table 11).

The results of the multiple comparison tests showed that the ROS scores were higher after the respondents were exposed to the thinned forest, and the difference was statistically significant (*p* < 0.05). However, even for the crowding forest, the scores were higher after the exposure, although there was no significant difference between the before-and-after scores. There were also no statistically significant differences in before and after exposure between the crowding and thinned forest settings (Table 12).

## 4. Discussion

### 4.1. Forest Environment, Impressions, and Restorative Effects

#### 4.1.1. Physical Environment

As shown in Table 4, the thinned forest showed a brighter environment with slightly wetter, windier, and louder conditions than those in the crowding forest.

#### 4.1.2. Impressions and Restorative Traits

As shown in Table 2 and Table 4, there were physical differences in the vegetation, including the undergrowth, between the crowding and thinned forests, depending on whether management had been conducted, despite the fact that originally the two had similar potential in vegetation. In this regard, by comparing the SD scale findings for the respondents’ impressions of the different forest settings, we confirmed more positive evaluations for the thinned forest. This result appeared to contradict the results of previous studies (i.e., Daniel [48]) but was consistent with our study hypothesis.

In addition, we found either statistically or marginally significant differences in the restorative traits like compatibility, coherence, and preference. These results suggest that the thinned forest had higher compatibility between appropriate activities, and we can also conclude that the thinned forest had greater coherence and was more preferred than the crowding forest. Taken together, the results of the present study suggest that the more restorative environment in the thinned forest was preferable to the environment in the crowding forest.

#### 4.1.3. Psychological Restorative Effects

We found no interactions among the POMS (feeling), PANAS (affect), and ROS (subjective restorativeness) indices. These results show that there were no unique restorative effects for feeling, affect, or subjective restorativeness or their combined influences depending on whether or not the forest had been thinned (condition difference) or between before and after exposure to the forest environment (time difference).

Then, we found no significant primary effects for any of the indices. This result shows that the presence or absence of forest management might not alone determine the psychological restorative effects of forests. It appears that forests themselves have restorative effects but that the quality of the effects differs depending on whether or not the forest is being managed. Conversely, we did observe significant effects for feeling (POMS: T–A, V, and C) and affect (PANAS: NA) and confirmed a marginally significant effect for the ROS. These results show that the study respondents felt improved feelings, NA, and subjective restorativeness just by being in the forests whether or not the forest had been managed.

### 4.2. Mutual Relationships

#### 4.2.1. Physical Environment Impressions and Restorative Traits

We observed large statistical differences in the respondents’ impressions depending on whether forest management had been carried out (see Table 5 and Table 6 for detailed scale differences). Overall, the respondents evaluated the thinned forest more highly than the crowding forest; however, because both the experiments were conducted on the same day, we believed that the differences in the presence or absence of management would be reflected in the respondents’ evaluations based on their five senses. For instance, for the “bright–dark” impression rating, the respondents rated the thinned forest as significantly brighter than the crowding forest, which could correspond to the physical difference due to forest management (i.e., the illumination intensity [lux] in the thinned forest setting was double that in the crowding forest setting).

However, for “quiet–noisy”, “warm–cool”, and “dry–wet”, we could not confirm any differences caused by the presence or absence of forest management, and we also observed no relationships between physical differences and respondents’ evaluations. That is, except for light, there were no differences in the respondents’ impressions between the thinned and crowding forests. As shown in Table 6, the respondents also rated compatibility and coherence as higher in the thinned forest setting. This implies that because thinned forests have been managed, it is easier to understand their traits, and they have greater restorative effects than crowding forests. These results show that in unmanaged forests, thinning can improve forest environments, which can improve users’ impressions and evaluations of forests. This consideration has been supported by several previous studies [40,42,43,45,48,49], which have reported that forest management results in higher (brighter, more preferable) evaluations because forest management brings a sense of ease to users; therefore, the results of this study can be interpreted as being consistent with previous studies. In addition, a previous study reported that traits of restorative environments could be improved by conducting forest management [66]. Takayama et al. [66] reported that when forests that are already being managed are thinned slightly, the forests’ traits are not influenced.

Initially, it may appear that the results of this study are inconsistent with those of Takayama et al. [66]. This could be because the forest these authors studied was already being maintained and was just thinned slightly, whereas we focused on investigating potential differences in restorative traits between managed and unmanaged forests. In other words, this study appears to be the first to scientifically suggest that forest management enhances restorative traits, especially compatibility and coherence, which can increase preferences for forest environments.

#### 4.2.2. Impressions and Psychological Restorative Effects

Based on the previous discussion, we considered the relationships between impressions and restorative traits and psychological restorative effects. We found statistically significant differences in the impressions and restorative trait evaluations between the conditions (thinned versus crowding forests); however, the indices of psychological effects, the POMS, PANAS, and ROS, did not show different effects between the conditions (Table 7, Table 9 and Table 11). We also made similar observations based on the results from the lower parts of Table 8, Table 10 and Table 12. The following factors could be considered reasons for our findings. First, we can suggest that viewing either forest setting would have been restorative because the respondents were not allowed to go deep into the forest for our experiment, and we can confirm this with the finding that the presence or absence of management (condition difference) yielded no significant primary effects based on the results of any of the indices we administered; however, we did observe that the time difference, that is, before or after the experiment, did influence T–A, V, and C from the POMS and NA from the PANAS, either significantly or marginally significantly.

In contrast, the results of the multiple comparisons we conducted of the index results (the upper parts of Table 8, Table 10 and Table 12) indicated that both conditions provided psychological restorative effects, although observing the details in depth, we did find greater restorative effects from the thinned forest than from the crowding one. The respondents rated the different forest environments differently in areas such as impressions and evaluations of the forests’ restorative traits. However, because there were no differences in the trait ratings, the differences in the evaluations were not particularly reflected in the psychological restorative effects. That is, whether or not the forests had been thinned, the respondents considered the forest environments to be restorative, but their ratings of the indicators appeared to increase when the forest was managed.

#### 4.2.3. Management and Psychological Restorative Effects

The physical indicators such as tree density and total basal area differed clearly between the thinned and crowding forests, and in general, we can perceive thinned forest environments to be bright, rich in diversity, well ventilated, and less humid (see Table 4 for details).

On the contrary, we observed no differences in the psychological restorative effects and indicators that have been confirmed to be influenced by forest management, with the exception of D from the POMS. In other words, we could not determine whether forest management greatly affected psychological restorative traits. Here referring to Takayama et al. [66], when we consider the assumption; stimulus (the Lazarus of the agency stress model [67]) → rating (determines the evaluation) → reaction relationship, the differences in forest environments due to the presence or absence of management, lead to differences in impressions and restorative traits (Table 5 and Table 6). However, we can suggest that ultimately, the differences in users’ evaluations do not affect the psychological restorative effects of forests (Table 7, Table 8, Table 9, Table 10, Table 11 and Table 12).

In other words, assuming the activity of sitting and viewing the forest landscape as in this study, we can evaluate that thinning forests increases their brightness, and eventually enhances user evaluations of the forests, forest management does not appear to dramatically increase the psychological restorative effects of viewing forests.

#### 4.2.4. Applying the Results

As discussed so far, forest management influences landscape appreciation but not the psychological restorative effects of the forests. This strongly suggests the need to improve forest environments based on user needs while maintaining low management costs. For example, if the planning focus is primarily to enhance the restorative effects, the focus within the forest should be on sidewalks and places to rest. Then, based on remaining funds, other factors can be prioritized in terms of managing the forest, making reasonable forest management possible. Separately, if we can demonstrate that maintaining restorative forest environments with lower costs and smaller labor forces, we could catalyze the restoration of abandoned forests, which would open up opportunities for more citizens to visit forests and experience their restorative effects on the mind and body, which could in turn decrease future medical costs. Moreover, the increase in forest users could increase people's concern about forests and drive more appropriate forest management strategies.

### 4.3. Limitations

In this study, while offsetting the sequential effects, we conducted experiments in both crowding (unmanaged) forests and thinned (managed) forests using 17 participants for both settings to compare our findings with those of similar psychological experiments conducted on-site [15,16,17,18,66]. We believe that our research has reasonably satisfied its validity as an empirical study. However, as this type of research always requires, we derived our conclusions from a limited number of subjects, and, separately, forest environments change greatly depending on the weather, season, time zone, and management status; therefore, it is necessary to pay attention when generalizing the results of this research. Moreover, to conduct this study, we prepared the crowding and thinned forests as experimental environments and attempted to conduct the two experiments under the same conditions except for the presence or absence of forest management. However, it is possible that we could not strictly control our environmental conditions as effectively as we could have in a laboratory (off-site), and our results might have been influenced by this as well as by other field (on-site) experiments. In addition, for this study, we investigated two research plots with similar forest types but different management techniques; however, because our experiment did not consist of actually thinning crowded forests, certain remarks are needed for interpreting the results of this research. Moreover, for our experiments, we placed our participants in comfortable chairs and had them relax while they viewed the forest environments, but people can conduct other activities in forests such as walking, trail running, and mountain biking. Therefore, future experiments should investigate the influence of management on more physical activities than just sitting.

### 4.4. Future Research

When forests are properly managed, their prospects will improve and feelings of depression or dejection also improve. For this result, as Takayama et al. [66] suggested, understanding will improve by referring to theories such as Appleton’s prospect and refuge theory [68] and Kaplan and Kaplan’s theory [64], which highlighted fear of natural environments and relaxation. To make forest environments comfortable, it is important for forest managers to have knowledge about the relationships between the environments and people’s senses of fear, unease, tension, and insecurity. This research revealed that forest management brings about positive effects on cognition and user evaluations and increase psychological restorative effects. As Appleton [68] noted, we also believe that our results were derived from the possibility that being able to see the forest well without being able to see the other respondents might have provided relief for our subjects.

However, in this study, because we chose primarily vigorous 30- to 40-year-old men as the respondents, it was possible that most of the men did not feel uneasy about the crowding forest (this tendency was confirmed by interviews we conducted after the experiment), in contrast to our research hypothesis, other theories, and cases in which women and people of other ages were respondents. (On the contrary, we did confirm significant differences in the respondents’ impressions of the crowding and thinned forests.) It appears conceivable to be a reason why only a small difference was obtained in the comparison of the psychological restorative effect in the crowding forest and the thinned forest. With reference to the results of this study, it is necessary to conduct additional research in laboratories using movies or photographs, more respondents, and respondents with other attributes.

## 5. Conclusions

In general, forest environments that provide Shinrin-yoku and recreational use are often considered problems in terms of trees in forest density management, promenade design, resting facilities, signboards, and pavilions from the beginning of their design, because forests appear to enhance users’ physical and psychological restorative effects and their experience of quality. However, forests consist of a variety of animals and plants, and in addition, forests with characteristic facilities degrade more quickly than urban areas because of the higher humidity. Accordingly, compared with other environments, it is very important to have not only an initial arrangement but also a method and planning for later management for proper maintenance. Here, considering the results of this research, the user impressions and restorative traits of forest environments show the differences caused by forest management; however, we can think of management plans based on our finding that psychological restorative effects will not differ greatly. For instance, when performing maintenance for Shinrin-yoku and recreational use, we could attempt to thoroughly manage all forests by conducting thinning and/or undergrowing. For all forests, the costs will increase in terms of expenses and manpower in terms of management and maintenance. Therefore, we suggest that it would be more useful to not attempt to perfectly manage all forests but to partially manage forests that users will not approach from forest roads or promenades, thereby reducing the maintenance costs and preserving the diversity of flora and fauna. If the environment changes in a certain forest, the diversity of the environment will increase in the whole forest area. Regarding the places where it is assumed that users intend to enter the forest from forest roads or to conduct active activities, it is necessary to manage forest environments with sharp focus, such as concentrating on maintenance. This increases the possibility of users’ experiencing a variety of forest environments and experiencing psychological restorative effects. Therefore, we will formulate an effective and cost-effective forest management plan.

### Summary

Finally, the findings obtained in this research can be summarized in the following three points:

(1) The impressions and evaluations of the restorative traits for the crowding versus thinned forest environments differed greatly, and the thinned forest were evaluated more positively than the crowding one.
(2) The differences in the physical environments between the crowding and thinned forests did not appear to be reflected in respondents’ impressions except for illuminance (on the SD scale).
(3) In terms of appreciating landscapes while sitting, it is possible that forest environments can bring about psychological restorative effects whether or not forest management is being conducted but that these effects can be partially enhanced by managing the forests.

## Figures and Tables

**Table 1:** Details of the research site.

		Crowding Forest	Thinned Forest
Location		Fuji Iyashinomori Woodland Study Center, Yamanaka Lake Town, Yamanashi Prefecture	
Area		0.25 ha (50 m × 50 m)	0.25 ha (50 m × 50 m)
Forest Status		Non managed mixed forest(80-year-old Larch and Dogwood )	well managed mixed forest(80-year-old Larch and Dogwood)
Weather			
26**–**29 July 2014	Day 1	Sunny	
	Day 2	Sunny	
	Day 3	Sunny (Partly cloudy)	
	Day 4	Sunny	
Monthly average temperature		21.7 °C	
Monthly average humidity		83.2%	

**Table 2:** Perspectives of the Crowding and Thinned Forest Settings.

		Crowding Forest	Thinned Forest
Stand density (number/ha)		1212 (*n* = 303)	1056 (*n* = 251)
Stand basal area (m^2^/ha)		32.7	44.3
Species composition (basal area; %)	Larch	66.5%	66.3%
	Dogwood	7.0%	10.1%
	Red pine	7.3%	7.3%
	Fir	0.0%	6.4%
	Japanese-alder	0.0%	1.8%
	Veitch’s silver fir	0.4%	1.8%
	Fuji cherry	0.3%	0.8%
	Maple	2.1%	0.6%
	Japanese wing nut	0.0%	0.6%
	Others	16.4%	4%
Hemispherical photograph		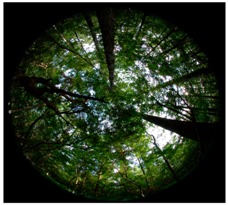	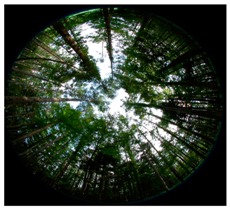
Scenery		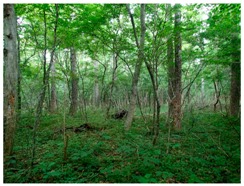	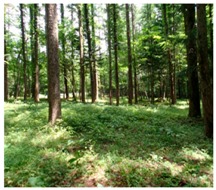

**Table 3:** Information on respondents.

	Number	Average (Age)	S.D. (Age)
Participants	17	40.2	±6.4
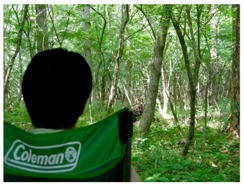		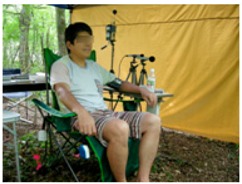	
Seeking at the landscape during experiment		State of respondent during experiment	

S.D., standard deviation.

**Table 4:** *T*-test results for the comparisons of the physical features of the crowding and thinned forests.

Item	Crowding Forest		Thinned Forest					
*n* = 125	Average	S.D.	Average	S.D.	*t*	*p*		*r*
Temperature (°C)	24.2	7.6	24.3	7.9	0.339	0.735	-	0.022
Relative Humidity (%)	70.0	34.4	73.3	39.3	4.240	0.000	**	0.260
Wind velocity (m/s)	0.23	0.06	0.13	0.03	3.873	0.000	**	0.238
Radiant heat (°C)	25.3	9.8	25.5	10.5	0.368	0.713	-	0.024
*n* = 72	Average	S.D.	Average	S.D.	*t*	*p*		*r*
Illuminance (lux)	119.2	48.5	255.0	97.6	14.700	0.000	**	0.664
Sound pressure (dB)	39.0	3.9	41.5	3.8	4.285	0.000	**	0.317

**: *p* < 0.01, *: *p* < 0.05, -: not significant, unpaired *t*-test; *n* = 125, *n* = 72.

**Table 5:** *T*-test results for the comparisons of the crowding and thinned forest settings using the SD scale.

	Crowding Forest		Thinned Forest					
	Average	S.D.	Average	S.D.	*t*	*p*		*r*
Bright (1)–Dark (7)	2.28	1.60	1.11	1.08	3.207	0.005	**	0.614
Open (1)–Closed (7)	2.44	1.72	1.11	1.18	2.576	0.020	*	0.53
Artificial (1)–Natural (7)	5.28	1.18	4.78	1.35	1.164	0.261	-	0.272
Smelly (1)–Odorless (7)	2.65	1.71	2.28	1.41	0.848	0.408	-	0.202
Still (1)–Animated (7)	2.94	1.30	3.44	1.38	1.231	0.235	-	0.287
Comfortable (1)–Uncomfortable (7)	2.39	1.65	1.11	0.96	2.537	0.021	*	0.525
Quiet (1)–Noisy (7)	2.00	1.61	1.50	1.38	1.231	0.235	-	0.287
Ugly (1)–Beautiful (7)	3.78	1.40	4.78	0.94	2.525	0.022	*	0.523
Pleasing sound (1)–Irritating noise (7)	1.94	1.26	2.06	1.16	0.270	0.790	-	0.066
Friendly (1)–Unfriendly (7)	2.06	1.35	1.33	1.03	1.959	0.067	#	0.43
Dull (1)–Refreshing (7)	3.78	1.77	4.89	1.08	2.149	0.046	*	0.463
Orderly (1)–Chaotic (7)	3.94	1.30	2.50	1.29	3.424	0.003	**	0.639
Warm (1)–Cool (7)	4.50	1.25	4.67	1.33	0.483	0.636	-	0.117
Insecure (1)–Secure (7)	3.72	1.64	4.89	1.02	2.817	0.012	*	0.565
Gentle lighting (1)–Too bright (7)	1.00	1.03	0.89	0.90	0.383	0.707	-	0.093
Thin (1)–Thick (7)	3.83	1.20	2.50	0.92	4.408	0.000	**	0.731
Flat (1)–Three dimensional (7)	4.39	1.38	4.44	1.34	0.212	0.834	-	0.052
Awaking (1)–Soothing (7)	3.89	1.32	3.94	1.21	0.152	0.881	-	0.037
Enchanted (1)–Disenchanted (7)	1.78	1.44	1.61	1.24	0.615	0.547	-	0.148
Fragrant (1)–Malodorous (7)	1.94	1.11	1.89	0.96	0.223	0.826	-	0.055
Non enjoyable (1)–Enjoyable (7)	4.06	1.55	4.89	1.18	1.815	0.087	#	0.403
Restless (1)–Calm (7)	4.28	1.41	5.06	0.87	2.072	0.054	#	0.45
Dry (1)–Wet (7)	3.11	1.08	3.17	0.92	0.148	0.884	-	0.036
Nondescript (1)–Unique (7)	2.61	1.20	3.00	1.41	1.197	0.248	-	0.279
Healthy (1)–Unhealthy (7)	1.94	1.47	1.11	0.83	2.557	0.020	*	0.528

**: *p* < 0.01, *: *p* < 0.05, #: *p* < 0.1, -: not significant, paired *t*-test; *n* = 18. Likert scale is seven stages, i.e., 1 (left item) and 7 (right item); For example, with regard to “Bright-dark” score, thinning forest seem to have lower score than crowding forest, however, as a result of subjective appraisal, it shows that thinned forests were evaluated brighter.

**Table 6:** *T*-test results for comparing the crowding and thinned settings using the PRS.

	Crowding Forest		Thinned Forest					
	Average	S.D.	Average	S.D.	*t*	*p*		*r*
Being away	35.9	8.82	38.8	7.98	1.483	0.156	-	0.339
Fascination	32.5	9.98	33.2	9.17	0.302	0.766	-	0.074
Coherence	18.3	6.12	22.2	6.35	2.044	0.057	#	0.445
Scope	24.2	8.83	26.3	7.58	1.155	0.264	-	0.27
Compatibility	26.6	8.15	32.1	7.52	2.190	0.043	*	0.47
Familiality	5.6	2.50	5.3	2.72	0.339	0.738	-	0.083
Preference	10.1	5.05	12.4	3.03	2.094	0.052	#	0.453

*: *p* < 0.05, #: *p* < 0.1, -: not significant, paired *t*-test; *n* = 18; PRS, Percieved restorativeness scale.

**Table 7:** Results for the two-way repeated-measures ANOVAs for the POMS scores.

POMS	Main Effect								Interaction			
	Condition Crowding vs. Thinned Forest				Time Pre vs. Post				Condition × Time			
	F	*p*		η^2^	F	*p*		η^2^	F	*p*		η^2^
Tension-Anxiety	0.455	0.505	-	0.009	7.094	0.012	**	0.062	0.243	0.625	-	0.003
Depression-Dejection	1.173	0.286	-	0.028	0.109	0.743	-	0.001	1.430	0.240	-	0.008
Anger-Hostility	0.570	0.456	-	0.012	0.172	0.681	-	0.002	0.564	0.458	-	0.006
Vigor	0.031	0.861	-	0.001	6.428	0.016	*	0.023	0.225	0.638	-	0.001
Fatigue	0.918	0.345	-	0.024	1.994	0.167	-	0.006	0.197	0.660	-	0.001
Confusion	0.792	0.380	-	0.018	5.265	0.028	**	0.034	0.015	0.903	-	0.001

**: *p* < 0.01, *: *p* < 0.05, -: not significant, Two-way repeated measures ANOVA; *n* = 18. POMS, Profile of mood states.

**Table 8:** Results of multiple comparisons of POMS scores for thinned versus crowding forests and for before and after forest exposure.

	**Crowding Forest**						**Thinned Forest**					
	**Pre**		**Post**				**Pre**		**Post**			
	**Average**	**S.D.**	**Average**	**S.D.**	***p***		**Average**	**S.D.**	**Average**	**S.D.**	***p***	
Tension–Anxiety	3.00	3.25	1.78	2.90	0.037	*	2.72	3.58	0.94	2.01	0.003	**
Depression–Dejection	0.71	1.27	1.00	1.57	0.136	-	0.50	1.54	0.33	0.84	0.393	-
Anger–Hostility	0.47	0.98	0.28	0.96	0.252	-	0.17	0.51	0.22	0.94	0.739	-
Vigor	4.94	4.95	6.89	5.99	0.005	**	5.56	5.54	6.89	5.81	0.047	*
Fatigue	2.18	2.79	1.94	2.71	0.340	-	1.56	1.95	1.11	1.97	0.072	#
Confusion	4.47	1.79	3.72	2.59	0.037	*	3.94	2.18	3.11	2.08	0.021	*
	**Pre**						**Post**					
	**Crowding Forest**		**Thinned Forest**				**Crowding Forest**		**Thinned forest**			
	**Average**	**S.D.**	**Average**	**S.D.**	***p***		**Average**	**S.D.**	**Average**	**S.D.**	***p***	
Tension–Anxiety	3.00	3.25	2.72	3.58	0.695	-	1.78	2.90	0.94	2.01	0.242	-
Depression–Dejection	0.71	1.27	0.50	1.54	0.517	-	1.00	1.57	0.33	0.84	0.040	*
Anger–Hostility	0.47	0.98	0.17	0.51	0.143	-	0.28	0.96	0.22	0.94	0.787	-
Vigor	4.94	4.95	5.56	5.54	0.643	-	6.89	5.99	6.89	5.81	1.000	-
Fatigue	2.18	2.79	1.56	1.95	0.276	-	1.94	2.71	1.11	1.97	0.146	-
Confusion	4.47	1.79	3.94	2.18	0.310	-	3.72	2.59	3.11	2.08	0.239	-

**: *p* < 0.01, *: *p* < 0.05, #: *p* < 0.1, -: not significant, ANOVA-Bonferroni; *n* = 18.

**Table 9:** Results for the two-way repeated-measures ANOVAs for the PANAS scores.

PANAS	Main Effect								Interaction			
	Condition Crowding vs. Thinned Forest				Time Pre vs. Post				Condition × Time			
	F	*p*		η^2^	F	*p*		η^2^	F	*p*		η^2^
Negative	0.018	0.895	-	0.003	6.692	0.014	*	0.053	0.359	0.553	-	0.003
Positive	0.530	0.472	-	0.014	0.064	0.801	-	0.007	0.064	0.801	-	0.001

*: *p* < 0.05, -: not significant, Two-way repeated measures ANOVA; *n* = 18. PANAS, Positive and negative affect schedule.

**Table 10:** Results of multiple comparison tests before and after exposure using the PANAS scores.

	**Crowding Forest**						**Thinned Forest**					
	**Pre**		**Post**				**Pre**		**Post**			
	**Average**	**S.D.**	**Average**	**S.D.**	***p***		**Average**	**S.D.**	**Average**	**S.D.**	***p***	
Negative	12.6	6.54	10.7	4.43	0.055	#	13.1	6.29	10.2	4.45	0.003	**
Positive	23.3	10.58	24.8	12.44	0.317	-	25.6	11.43	27.8	12.20	0.137	-
	**Pre**						**Post**					
	**Crowding Forest**		**Thinned Forest**				**Crowding Forest**		**Thinned Forest**			
	**Average**	**S.D.**	**Average**	**S.D.**	***p***		**Average**	**S.D.**	**Average**	**S.D.**	***p***	
Negative	12.6	6.54	13.1	6.29	0.672	-	10.7	4.43	10.2	4.45	0.844	-
Positive	23.3	10.58	25.6	11.43	0.562	-	12.4	24.78	27.8	12.20	0.445	-

**: *p* < 0.01, #: *p* < 0.1, -: not significant, ANOVA-Bonferroni; *n* = 18.

**Table 11:** Results for the two-way repeated-measures ANOVAs for the ROS scores.

ROS	Main Effect								Interaction			
	Condition Crowding vs. Thinned Forest				Time Pre vs. Post				Condition × Time			
	F	*p*		η^2^	F	*p*		η^2^	F	*p*		η^2^
	0.021	0.886	-	0.001	3.337	0.077	*#*	0.034	0.0544	0.817	-	0.001

#: *p* < 0.1, -: not significant, Two-way repeated measures ANOVA; *n* = 18. ROS, Restorative outcome scale.

**Table 12:** Results for the multiple comparison tests before and after exposure using ROS scores.

**Crowding Forest**						**Thinned Forest**					
**Pre**		**Post**				**Pre**		**Post**			
**Average**	**S.D.**	**Average**	**S.D.**	***p***		**Average**	**S.D.**	**Average**	**S.D.**	***p***	
29.61	6.68	31.89	7.68	0.12	-	29.6	6.35	32.50	7.94	0.05	*
**Pre**						**Post**					
**Crowding Forest**		**Thinned Forest**				**Crowding Forest**		**Thinned Forest**			
**Average**	**S.D.**	**Average**	**S.D.**	***p***		**Average**	**S.D.**	**Average**	**S.D.**	***p***	
29.61	6.68	29.56	6.35	0.974	-	31.89	7.68	32.50	7.94	0.720	-

*: *p* < 0.05, -: not significant, ANOVA-Bonferroni; *n* = 18.

## Supplementary Materials

The following are available online at www.mdpi.com/1660-4601/14/7/800/s1, all data in this research.
